# Acute Angiographic and Intermediate-Term Clinical Results of Patients with Non-Left Main Coronary Bifurcation Lesions Treated with BVS by Jailed Semi-Inflated Balloon Technique and Provisional Side-Branch Stenting Strategy

**DOI:** 10.1155/2019/9896267

**Published:** 2019-10-03

**Authors:** Chieh-Shou Su, Keng-Hao Chang, Chih-Hung Lai, Yu-Wei Chen, Tzu-Hsiang Lin, Hung-Chih Pan, Tsun-Jui Liu, Wen-Lieng Lee

**Affiliations:** ^1^Cardiovascular Center, Taichung Veterans General Hospital, Taichung, Taiwan; ^2^Institute of Clinical Medicine, Department of Medicine, National Yang-Ming University School of Medicine, Taipei, Taiwan; ^3^Department of Internal Medicine, Cheng Ching Hospital, Taichung, Taiwan; ^4^Division of Cardiology, Asia University Hospital, Taichung, Taiwan; ^5^Department of Medicine, National Yang Ming University School of Medicine, Taipei, Taiwan

## Abstract

**Background:**

To evaluate the acute angiographic and intermediate-term clinical results of patients with non-left main (LM) coronary artery bifurcation disease (CABD) treated with BVS, as compared with those treated with DES, using the jailed semi-inflated balloon technique (JSIBT) for side branch (SB) protection and provisional stenting.

**Methods and Results:**

Sixty-eight patients with non-LM CABD who had undergone provisional one-stent implantation with SB protection by JSIBT between January 2015 and December 2017 were retrospectively enrolled. Among them, 20 patients received Absorb BVS implantation and 48 patients received DES implantation. Patients in the BVS group were younger and had higher BMI, total cholesterol, low-density lipoprotein cholesterol, and hemoglobin but had lower serum creatinine and lower prevalence of prior PCI and MI. No SB balloon rupture/entrapment occurred in either group. The incidence of SB dissection/occlusion and SB in need of rewiring or stenting was rare in both groups and showed no significant difference between them. Postinterventional TIMI flow significantly increased in both groups. The intermediate-term clinical outcomes were good in terms of incidence of target lesion failure, target lesion revascularization, target vessel revascularization, myocardial infarction, and all-cause death in both groups.

**Conclusion:**

The use of JSIBT for treating CABD with modern BVS can provide SB protection as similar as those with DES, even with higher incidence of acute SB dissection/occlusion. The immediate angiographic results and acute and intermediate-term clinical outcomes were also similar in both groups. Our study results demonstrate that JSIBT might be a safe and alternative SB protection tool for BVS in patients with complex CABD.

## 1. Introduction

Coronary artery bifurcation disease (CABD) occurs in 15–20% of coronary artery disease (CAD) patients undergoing percutaneous coronary intervention (PCI) [1, 2] and remains a considerable challenge in clinical practice despite advances in modern interventional techniques and stents. Currently, the provisional side branch (SB) stenting strategy is considered the standard practice for most CABD [3–5]. A protection guidewire placed inside the SB prior to the main vessel (MV) stenting remains the minimal requirement for CABD PCI. However, there remains a risk of acute SB occlusion after MV stenting, especially in a true bifurcation lesion with large plaque burden, very tight stenosis at the SB ostium, diminished baseline SB blood flow, or very blunt bifurcation angulations [6, 7]. The jailed balloon technique (JBT) and jailed semi-inflated balloon technique (JSIBT) have been introduced to reduce acute SB occlusion at the time of the MV drug-eluting stent (DES) implantation [7–11]. Bioresorbable vascular scaffold (BVS) was introduced and provides an alternative choice for certain patients with bifurcation lesions. In consideration of the thicker stent struts and higher post dilatation pressure, how to preserve the SB might be a major concern. To date, no studies have addressed the use of JSIBT in patients treated with BVS for complex CABD. We therefore undertook the current study to investigate the acute angiographic and clinical results as well as the intermediate-term outcomes of patients treated with BVS and JSIBT for complex CABD as compared with those treated with DES at our institution.

## 2. Methods

### 2.1. Study Population

Patients with non-LM CABD who underwent provisional one-stent strategy with SB protection by JSIBT between January 2015 and December 2017 were retrospectively enrolled and analyzed. Patients with heavily calcified lesions demanding rotablator atherectomy, an SB vessel size of ≤1.5 mm diameter, or cardiogenic shock/arrest on admission were excluded. Each CABD was classified according to the Medina classification, which defines Medina (1.1.1), (1.0.1), and (0.1.1) lesions as true bifurcation lesions. Written informed consent for PCI was obtained from all patients. The baseline demographic data, interventional details, and in-hospital and intermediate-term outcomes were retrospectively reviewed in detail using medical records in the hospital database and then were statistically analyzed. The study protocol was reviewed and approved by the Institutional Review Board/Ethics Committee of Taichung Veterans General Hospital, Taichung, Taiwan.

### 2.2. Intervention Procedures

**Figure 1:**
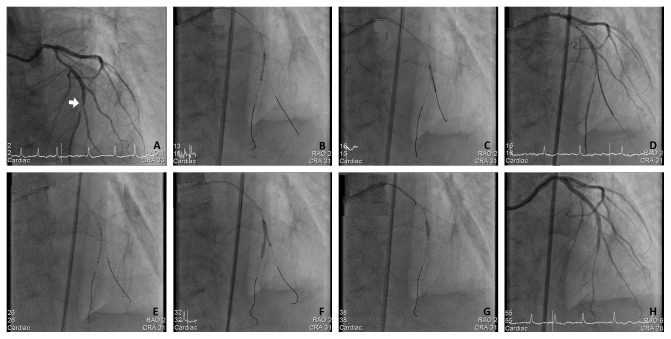
The steps of drug-eluting stent (DES) implantation for a complex bifurcation lesion using the jailed semiinflated balloon technique (JSIBT). (a) Diagnostic coronary angiography (CAG) at anteroposterior and cranial 33° projection showed a true distal left anterior descending (LAD) artery bifurcation lesion (white arrow, Medina classification 1.1.1). (b) Wiring of the main vessel (MV) LAD and side branch (SB), and balloon dilatation of the MV. (c) Balloon dilatation of the diagonal side branch (SB). (d) CAG postballoon dilatation of both the MV and SB revealed significant stenosis of both branches. (e) Placement of a semicompliant balloon in the diagonal SB and DES in distal LAD, covering the MV lesion. (f) DES and a semicompliant balloon were inflated simultaneously. The SB balloon was inflated at low pressure (6 atmospheres) and DES at less than nominal pressures. (g) For optimization of the MV stent, the proximal optimal technique was performed with a short noncompliant balloon. (h) Final CAG at LAO 6° and Cranial 28° projection showed a good angiographic result and bifurcation flow.

**Figure 2:**
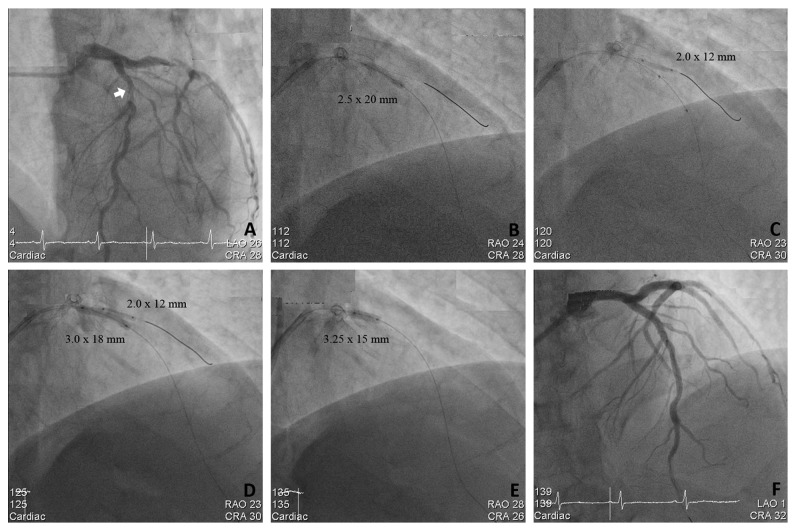
The steps of bioresorbable vascular scaffolding (BVS) implantation for a complex bifurcation lesion using the jailed semiinflated balloon technique (JSIBT). (a) Diagnostic coronary angiography (CAG) at LAO 26° and caudal 28° projection showed a true proximal left anterior descending (LAD) artery bifurcation lesion (Medina classification 1.1.1). (b) Wiring of the main vessel (MV) LAD and side branch (SB) and balloon dilatation of the MV. (c) Balloon dilatation of the diagonal SB and BVS was advanced to middle of the MV. (d) JSIBT with simultaneous inflation of BVS and a semicompliant balloon. The SB balloon is inflated to a low pressure (4 atmospheres) and BVS less than nominal pressures. (e) For optimization of the MV scaffold, the proximal optimal technique was performed with a short noncompliant balloon. (f) Final CAG at LAO 1° and cranial 32° projection showed a good angiographic result and bifurcation flow.

All procedures were carried out using the standard PCI protocols of our cath lab. The patients received a loading dose of aspirin (300 mg) and clopidogrel (300–600 mg) or ticagrelor (180 mg) prior to or at the time of PCI. Every patient received anticoagulation using heparin during the procedure with targeted ACT of 300″, while use of glycoprotein IIb/IIIa inhibitors were left to the operator's discretion. The procedure of JSIBT for CABD is described in detail elsewhere [11, 12]. The steps of JSIBT applied to our patients are shown in Figure 1 (JSIBT with DES) and Figure 2 (JSIBT with BVS). The brands of DES implanted for these CABD patients were chosen by the operator discretion, and the brand of BVS implanted was Abbott BVS (Absorb, Abbott Vascular). Both the SB and MV were wired, and then the bifurcation lesions were predilated by a one quarter-size smaller balloon or one of an equal size for both the MV and SB. Then the DES or BVS was advanced into the MV and placed overriding the bifurcation lesion. Thereafter, a semicompliant quarter-size smaller balloon or one of an equal size was advanced into the SB beforehand, making sure that the proximal portion of the balloon 1-2 mm protruded into the MV. The protection balloon in the SB was inflated at low pressure (usually 6–8 atm), and subsequently the MV DES or BVS balloon was deployed slowly at subnominal pressure, jailing the semi-inflated SB balloon. The SB balloon was kept inflated during the MV DES or BVS implantation. The DES balloon inflation was maintained for about 20 seconds, and the BVS balloon was kept inflated for about 30–40 seconds. Both the SB balloon and the MV DES or BVS balloon were deflated at the same time and the SB balloon was removed. The MV balloon was then reinflated at nominal pressure to restore the deformed stent or the scaffold and fully expand the stent or the scaffold. In the final step, postdilatation of the whole stent/scaffold with a noncompliant balloon and proximal optimal dilatation therapy (POT) of the stent/scaffold segment were performed to achieve good stent/scaffold apposition to the MV wall. No rewiring of the SB was done if angiography showed SB patency. However, in the event of acute occlusion or imminent jailing, the SB was rewired and the kissing balloon technique (KBT) was completed in order to restore SB flow. Intravascular ultrasound (IVUS) was performed on a case-by-case basis during the procedure to optimize the angiographic results.

### 2.3. Definition of Study Endpoints

The primary study endpoints are in-hospital death, target lesion revascularization (TLR), and target vessel revascularization (TVR), and the secondary study endpoints are myocardial infarction (MI), target lesion failure (TLF), and all-cause death. TLR is defined as any repeat percutaneous intervention of the target lesion or bypass surgery of the target vessel performed for restenosis or other complication of the target lesion. TVR is defined as any repeat percutaneous intervention or surgical bypass of any segment of the target vessel. MI is diagnosed by the criteria of universal definition [13] during the follow-up period. TLF is defined as the combination of cardiac death, target vessel MI, or clinically driven TLR. Any revascularization is defined as any repeat percutaneous intervention or bypass surgery for restenosis of the target lesion or de novo lesion(s) of the target vessel or non-target vessel.

### 2.4. Statistical Analysis

Continuous variables are presented as median with interquartile range because of nonnormally distributed variables. Categorical variables are presented as numbers and percentages. Continuous variables of the two groups were analyzed by the Mann–Whitney *U* test. Categorical variables were analyzed by the Chi-square test or Fisher exact test. Pre- and postprocedure quantitative coronary angiography (QCA) analyses were compared using the Wilcoxon signed-rank test in each group. A *P* value of less than 0.05 was considered statistically significant. All statistical analyses were performed using SPSS 19.0 (SPSS Inc., Chicago, Illinois, USA) software.

## 3. Results

### 3.1. Baseline Characteristics of Patients with Non-LM CABD Who Underwent DES/BVS Implantation Utilizing JSIBT

Between January 2015 and December 2017, a total of 68 patients with CABD treated with provisional stenting strategy using JSIBT were enrolled. The baseline characteristics of all study patients are shown in Table 1. Among them, 20 patients were treated with BVS, and the remainder (*N* = 48) were treated with DES. Patients in the BVS group were younger, had higher BMI, total cholesterol, low-density lipoprotein cholesterol, and hemoglobin but had lower serum creatinine as well as a lower prevalence of prior PCI and MI compared with the DES group. The distribution of gender, background risk profiles (HTN, DM, dyslipidemia with statin therapy, and smoking), clinical presentations and diagnosis, severity of CAD, and characteristics of bifurcation lesion were all similar between the two groups.

### 3.2. Angiographic and Interventional Characteristics of JSIBT for Non-LM CABD

Angiographic and procedural characteristics are shown in Table 2, and QCA analysis for the MV and SB at the baseline and postprocedure are shown in Table 3. More patients received PCI using the 7 Fr guide catheter in the BVS group, but the 6 Fr one in the DES group. The transfemoral approach was more frequently used in the DES group (29.2% versus 0% and *P*=0.007). In the BVS group, the MV stent was larger in size and shorter in length compared with that used in the DES group, but the size and length of the SB protection balloon were similar to those in the DES group. POT was performed in all patients in both groups, but the balloon size was larger in the BVS group compared to that in the DES group. No SB balloon rupture or entrapment occurred in either groups. Four patients in the BVS group and four patients in the DES group had SB dissection, 2 and 9 patients in need of SB rewiring and 0 and 2 patients demanding SB stenting. However, these differences were not significant between the two groups. Postinterventional thrombolysis in myocardial infarction (TIMI) blood flow was significantly increased as compared to the preinterventional TIMI flow in both the BVS and DES groups. However, pre- and postinterventional TIMI blood flows did not show significant differences between the two groups. The MLD and stenosis severity improved in the proximal and distal ends of the MV and SB following PCI in both the BVS and DES groups.

### 3.3. In-Hospital and Out-of-Hospital Clinical Outcomes

The patients' clinical outcomes are shown in Table 4. The incidences of in-hospital death were similar between the two groups. There was one mortality in the DES group related to an accidental injury leading to massive subdural hematoma in the restroom. Despite emergent surgery to remove the hematoma, she passed away 7 days later.

Clinical follow-ups were available for all patients with a median follow-up period of 1.8 and 1.3 years in the BVS and DES groups, respectively. Four and sixteen patients received angiographic follow-up in the BVS and DES groups respectively. The incidence of TLF, TLR, TVR, MI, and all-cause death were similar between the two groups.

## 4. Discussion

The current study demonstrated that use of JSIBT as a novel SB protection method in complex non-LM CABD interventions treated with BVS provided effective SB protection and good acute procedural outcomes and intermediate-term clinical outcomes equivalent to benefits conferred by JSIBT in bifurcation lesions treated by DES. Despite the greater thickness of BVS struts and the greater risk of SB loss, JSIBT can offer the same degree of protection for the SB in CABD patients treated with BVS as that of DES.

CABD occurs in 15–20% of CAD patients undergoing PCI, [1, 2] remains technically challenging, and is also associated with a high rate of procedural complication and adverse cardiovascular outcomes even in the DES era [10, 14–18]. PCI of CABD is associated with greater prevalence rates of SB occlusion and periprocedural MI as well as poorer clinical outcomes in terms of TLR and stent thrombosis (ST). The complex anatomy and dynamic nature of bifurcation lesions make them prone to plaque or carina shift, change in bifurcation angles, vessel spasm, and/or SB dissection/occlusion during PCI. Currently, the one-stent strategy with provisional SB stenting is considered the preferred approach for most CABD, [3–5] but has also been associated with a significant risk of SB compromise and periprocedural MI [6, 19, 20]. Novel methods are needed to reduce SB events in bifurcation lesion interventions using the provisional one-stent strategy.

In order to protect the SB during PCI of CABD, the jailed wire technique, JBT, and JSIBT have been applied and have given rise to various standards or novel SB protection techniques [7–11, 21]. The JSIBT, an extension of the jailed wire technique and JBT, was first introduced in 2015 by Çaylı et al. [11, 22] who performed the provisional one-stent strategy with JSIBT for CABD for 148 lesions in 137 patients. Among these patients, 64.2% had ACS and 73.7% had true bifurcation lesions. TIMI 3 blood flow of both MV and SB after MV stenting was 100%, with no SB occlusion, no rupture or entrapment of the inflated balloon, and the clinical outcomes after MV DES treatment in terms of in-hospital stay and one-month follow-up without a composite of cardiac death, myocardial infarction, or target lesion revascularization were excellent. This novel technique provided an amazing way to protect the SB during CABD intervention using the provisional one-stent strategy even though 2.7% of patients presented with dissection of the SB ostium, and 2.0% of patients needed SB stenting with final KBT.

BVS was recently introduced as a novel coronary stent system, intended to potentially reduce the long-term limitation of metallic stents, such as permanent vessel caging, permanent SB jailing, or impairment of vasomotion [22]. Interventional cardiologists around the world soon embraced this concept in clinical practice. However, BVS implantation was not recommended for CABD due to the use of thick stent struts and lack of proper SB protection methods. Various approaches have since been introduced to solve this issue, [23] such as the provisional one-stent strategy with SB balloon dilatation, [24] sequential balloon dilatation, kissing balloon technique [25] or two-stent strategy with culotte, and [26] mini-crush or T-stenting technique [27]. All of the aforementioned approaches originated and were modified from techniques used in the DES era. To the best of our knowledge, our study is the first to test the feasibility and efficacy of JSIBT for SB protection in cases treated with BVS and one-stent strategy for complex CABD. We achieved a TIMI 3 final flow of 100% in both the MV and SB after MV BVS with significant postprocedural improvement as compared to that measured preprocedurally. Despite the use of thicker scaffold struts and the greater risk of SB events, there was no SB occlusion, rupture, or entrapment of the jailed semiinflated balloon, and the clinical outcomes during hospital stay and at a median of 1.8 years' follow-up were excellent without major adverse cardiac events in terms of in-hospital death, TLF, TLR, TVR, MI, and all-cause death. Nonetheless, 20% of patients presented with SB ostium dissection, and 10% of patients required SB rewiring and sequential balloon dilatation during the procedure. This might have been caused by the larger scaffold size and high inflating pressure of our jailed balloon in the SB. Otherwise, SB protection using JSIBT in our study demonstrated 8.3% of SB dissection and 4.1% of SB stenting needed in the DES arm with similar SB balloon inflation pressure in comparison to that in the BVS arm. As compared to our DES arm using the same strategy, there was no significant difference in interventional outcomes, i.e., SB protection and clinical outcomes, in the short and intermediate terms. In summary, JSIBT could be safely and effectively applied in BVS treatment of complex CABD.

## 5. Limitations

This study has some limitations. Firstly, this was a nonrandomized, retrospective, observational, case-cohort study and therefore subject to all the limitations inherent in the study design. Secondly, the study population in both groups was relatively small. As JSIBT is a new concept in the treatment of CABD (it was introduced in 2015 by Çaylı et al. [11]), we limited the application of this technique for the treatment of complex CABD in which SB may not be safe-guarded by other approaches. Furthermore, Abbott Absorb BVS was first made available in our hospital in late 2015. The aforementioned reasons explain why a relatively small number of patients were treated with this technique in our cath lab. However, our results demonstrate that JSIBT is not only useful for DES but is also applicable to different stent platforms. Our results are encouraging, and we believe that this protection strategy has potential for application to other bioresorbable scaffolds (BRS) in the market as well as to those currently undergoing trials. Thirdly, imaging studies, especially optical coherence tomography (OCT), have been shown to be useful for evaluation of BVS and SB structures immediately after implantation and for assessing long-term neointimal coverage and scaffold resorption. These imaging studies, however, were not performed in the current study. Nonetheless, the excellent long-term outcomes and lack of MACE demonstrate that JSIBT is a feasible, practical, and an effective method of protecting the SB in BVS treatment of complex CABD.

## 6. Conclusion

The use of JSIBT for treating complex CABD with the modern BVS, as compared to DES, was shown to provide excellent SB protection and maintain SB blood flow with very low incidence of acute SB dissection/occlusion. The acute- and intermediate-term clinical outcomes were excellent as well. Our study results confirm that JSIBT is also a safe and effective SB protection approach for BVS treatment of complex CABD. As this is a small study, further large-scale studies with imaging studies and long-term clinical follow-up data are warranted to confirm our findings and their clinical value.

## Figures and Tables

**Table 1:** Demographic characteristics of all coronary bifurcation lesion patients receiving PCI, utilizing the jailed semiinflated balloon technique.

	BVS (*N* = 20)	DES (*N* = 48)	*P* value
Gender M/F (*N*, %)	18/2 (90/10)	44/4 (92/8)	0.827
Age (years)	58.5 (47.3, 61.8)	69.0 (55.3, 75.3)	<0.001
Hypertension (*N*, %)	18 (90.0)	42 (87.5)	0.772
Diabetes mellitus (*N*, %)	8 (40.0)	27 (56.2)	0.222
Statins for dyslipidemia (*N*, %)	16 (80.0)	41 (85.4)	0.719
Smoking (*N*, %)	12 (60.0)	32 (66.7)	0.600
Prior PCI (*N*, %)	0 (0)	24 (50.0)	<0.001
Prior MI (*N*, %)	0 (0)	16 (33.3)	0.003
Prior CABG (*N*, %)	0 (0)	2 (4.2)	0.358
Admission diagnosis (*N*, %)			0.364
STEMI	2 (10.0)	6 (12.5)	
UAP/NSTEMI	4 (20.0)	17 (35.4)	
SCAD	14 (70.0)	25 (52.1)	
BMI (kg/m^2^)	29.7 (26.4, 32.8)	26.2 (23.5, 30.1)	0.006
Hemoglobin (mg/dl)	14.9 (13.3, 15.8)	13.9 (11.1, 15.2)	0.037
Total cholesterol (mg/dl)	159.5 (143.0, 187.0)	138.5 (122.5, 196.0)	0.022
LDL-C (mg/dl)	91.5 (83.0, 120.0)	78.5 (64.5, 101.5)	0.031
BUN (mg/dl)	17.0 (15.0, 20.0)	17.5 (14, 24.8)	0.646
Creatinine (mg/dl)	0.89 (0.82, 0.91)	0.93 (0.86, 1.25)	0.008
LVEF (%)	60.0 (49.0, 60.0)	53.5 (42.0, 59.0)	0.117
Severity of CAD			
Vessel numbers (*N*)	2 (1, 3)	1.5 (1, 2.75)	0.767
MVD (*N*, %)	12 (60.0)	24 (50.0)	0.452
Syntax score	15 (12, 29)	19 (13.6, 25.1)	0.571
Left main disease (*N*, %)	0 (0)	3 (6.3)	0.256
Bifurcation lesion			
Location			0.539
LAD (*N*, %)	16 (80.0)	35 (72.9)	
LCX (*N*, %)	4 (20.0)	13 (27.1)	
RCA (*N*, %)	0 (0)	0 (0)	
Medina classification (*N*, %)			0.445
1.1.1	16 (80.0)	28 (58.3)	
1.0.1	0 (0)	6 (12.5)	
0.1.1	0 (0)	8 (16.7)	
1.1.0	4 (20.0)	6 (12.5)	
0.0.1	0 (0)	0 (0)	
1.0.0	0 (0)	0 (0)	

Data are presented as median (interquartile range) for continuous variables and *N* (%) for categorical variables. The Chi-square or Fisher exact test for categorical variables and the Mann–Whitney *U* test for continuous variables. BVS, bioresorbable vascular scaffold; DES, drug-eluting stent; PCI, percutaneous coronary intervention; MI, myocardial infarction; CABG, coronary artery bypass grafting; STEMI, ST-segment elevation myocardial infarction; UAP, unstable angina pectoris; NSTEMI, non-ST segment elevation myocardial infarction; SCAD, stable coronary artery disease; BMI, body mass index; LDL-C, low-density lipoprotein cholesterol; LVEF, left ventricular ejection fraction; CAD, coronary artery disease; MVD, multiple vessel disease; LAD, left anterior descending artery; LCX, left circumflex artery; RCA, right coronary artery.

**Table 2:** Interventional characteristics of the jailed semiinflated balloon technique.

	BVS (*N* = 20)	DES (*N* = 48)	*P* value
Guide size			0.089
6 (*N*, %)	8 (40.0)	30 (62.5)	
7 (*N*, %)	12 (60.0)	18 (37.5)	
Approach (radial/femoral) (*N*, %)	20/0 (100/0)	34/14 (70.8/29.2)	0.007
MV stent			
Size (mm)	3.25 (3.0, 3.5)	2.75 (2.75, 3.0)	<0.001
Length (mm)	20 (18, 23)	30 (19, 38)	0.002
SB lesion length (mm)	12.5 (8.9, 17.2)	10.7 (8.0, 19.0)	0.936
SB balloon			
Size (mm)	2 (2, 2)	2 (2, 2.5)	0.592
Length (mm)	12.0 (12.0, 20.0)	12.0 (12.0, 20.0)	0.728
Inflation pressure (atm)	6.5 (6.0, 8.0)	6 (6.0, 8.0)	0.926
Proximal optimal dilatation (*N*, %)	20 (100)	48 (100)	—
BC size (mm)	3.25 (3.0, 3.5)	3.0 (2.75, 3.25)	0.003
Kissing balloon technique (*N*, %)	0 (0)	9 (18.8)	0.050
SB complication			
Dissection (*N*, %)	4 (20.0)	4 (8.3)	0.221
Occlusion (*N*, %)	0 (0)	0 (0)	―
SB balloon rupture/entrapment (*N*, %)	0 (0)	0 (0)	—
SB rewiring (*N*, %)	2 (10.0)	9 (18.8)	0.487
SB stenting (*N*, %)	0 (0)	2 (4.1)	0.358
Preinterventional TIMI flow			
MV TIMI flow			
Median (Q25, Q75)	3 (2, 3)	3 (1.25, 3)	0.577
TIMI 0 (*N*, %)	0 (0)	6 (12.5)	
TIMI 1 (*N*, %)	0 (0)	6 (12.5)	
TIMI 2 (*N*, %)	8 (40)	6 (12.5)	
TIMI 3 (*N*, %)	12 (60)	30 (62.5)	
SB TIMI flow			
Median (Q25, Q75)	3 (2, 3)	3 (2, 3)	0.946
TIMI 0 (*N*, %)	0 (0)	2 (41.7)	
TIMI 1 (*N*, %)	2 (10)	2 (41.7)	
TIMI 2 (*N*, %)	4 (20)	10 (20.8)	
TIMI 3 (*N*, %)	14 (70)	34 (70.8)	
Postinterventional TIMI flow			
MV TIMI flow			
Median (Q25, Q75)	3 (3, 3)^*∗*^	3 (3, 3)^‡^	1.000
TIMI 0 (*N*, %)	0 (0)	0 (0)	
TIMI 1 (*N*, %)	0 (0)	0 (0)	
TIMI 2 (*N*, %)	0 (0)	0 (0)	
TIMI 3 (*N*, %)	20 (100)	48 (100)	
SB TIMI flow			
Median (Q25, Q75)	3 (3, 3)^†^	3 (3, 3)^§^	1.000
TIMI 0 (*N*, %)	0 (0)	0 (0)	
TIMI 1 (*N*, %)	0 (0)	0 (0)	
TIMI 2 (*N*, %)	0 (0)	0 (0)	
TIMI3 (*N*, %)	20 (100)	48 (100)	

Data are presented as median (interquartile range) for continuous variables and *n* (%) for categorical variables. The Chi-square or Fisher exact test for categorical variables and a Mann-Whitney *U* test for continuous variables. ^*∗*^^,†,‡,§^Analyses of pre- and post-procedure TIMI flow were compared using the Wilcoxon signed-rank test in each group. BVS, bioresorbable vascular scaffold; DES, drug-eluting stent; MV, main vessel; SB, side branch; BC, balloon catheter; TIMI, thrombolysis in myocardial infarction.

**Table 3:** Quantitative coronary angiographic analysis of the jailed semiinflated balloon technique.

	BVS (*N* = 20)			DES (*N* = 48)		
	Baseline	Postprocedure	*P* value^a^	Baseline	Postprocedure	*P* value^b^
Proximal main vessel						
RVD (mm)	3.3 (3.0, 3.3)	3.4 (3.2, 3.7)	0.001	3.1 (2.8, 3.3)	3.2 (3.0, 3.5)	<0.001
MLD (mm)	0.9 (0.4, 1.0)	2.8 (2.6, 3.1)	<0.001	0.8 (0.5, 1.1)	3.0 (2.9, 3.2)	<0.001
Diameter stenosis (%)	74.6 (68.8, 89.7)	17.5 (16.1, 25.1)	<0.001	73.8 (61.5, 81.3)	6.0 (2.3, 10.2)	<0.001
Distal main vessel						
RVD (mm)	2.6 (2.5, 2.7)	2.9 (2.7, 3.1)	0.001	2.5 (2.2, 2.7)	2.9 (2.8, 3.0)	<0.001
MLD (mm)	1.0 (0.9, 1.2)	2.7 (2.6, 3.0)	<0.001	0.8 (0.6, 0.9)	2.8 (2.6, 3.0)	<0.001
Diameter stenosis (%)	57.7 (51.5, 66.7)	6.1 (4.7, 11.4)	<0.001	69.5 (65.5, 75.0)	4.8 (3.2, 7.6)	<0.001
Side branch						
RVD (mm)	1.9 (1.8, 2.2)	2.1 (2.0, 2.3)	0.001	2.1 (1.8, 2.3)	2.2 (2.0, 2.3)	<0.001
MLD (mm)	0.8 (0.6, 0.9)	1.9 (1.7, 2.0)	<0.001	0.7 (0.4, 1.0)	1.9 (1.7, 2.1)	<0.001
Diameter stenosis (%)	58.1 (43.9, 68.3)	16.2 (5.7, 22.7)	<0.001	67.5 (56.3, 78.2)	10.8 (5.0, 17.4)	<0.001

Continuous variables of the two groups were analyzed by the Mann–Whitney test; ^a,b^baseline and postprocedure quantitative coronary angiography analyses were compared using the Wilcoxon signed-rank test. BVS, bioresorbable vascular scaffold; DES, drug-eluting stent; RVD, reference vessel diameter; MLD, minimal lumen diameter.

**Table 4:** In-hospital and out-of-hospital clinical outcomes of patients with coronary bifurcation lesions receiving PCI, utilizing the jailed semiinflated balloon technique.

	BVS (*N* = 20)	DES (*N* = 48)	*P* value
Median clinical follow-up years	1.8 (1.6, 2.5)	1.3 (0.8, 1.8)	0.001
Angio follow-up (*N*, %)	4 (20.0)	16 (33.3)	0.250
In-hospital death (*N*, %)	0 (0)	1 (2.1)	0.519
TLF (*N*, %)	0 (0)	0 (0)	—
TLR (*N*, %)	0 (0)	0 (0)	—
TVR (*N*, %)	0 (0)	4 (8.3)	0.182
MI (*N*, %)	0 (0)	0 (0)	—
All-cause death (*N*, %)	0 (0)	3 (6.3)	0.251

Data are presented as median (interquartile range) for continuous variables and *N* (%) for categorical variables. The Chi-square or Fisher exact test for categorical variables and the Mann-Whitney *U* test for continuous variables. BVS, bioresorbable vascular scaffold; DES, drug-eluting stent; TLF, target lesion failure; TLR, target lesion revascularization; TVR, target vessel revascularization; MI, myocardial infarction.

## Data Availability

We are not allowed to share original study data publicly because of the hospital and institution policies, but they are available from the corresponding author on reasonable request.

## References

[1] Myler R. K., Shaw R. E., Stertzer S. H. (1992). Lesion morphology and coronary angioplasty: current experience and analysis. *Journal of the American College of Cardiology*.

[2] Iakovou I., Ge L., Colombo A. (2005). Contemporary stent treatment of coronary bifurcations. *Journal of the American College of Cardiology*.

[3] Brunel P., Lefevre T., Darremont O., Louvard Y. (2006). Provisional T-stenting and kissing balloon in the treatment of coronary bifurcation lesions: results of the French multicenter “TULIPE” study. *Catheterization and Cardiovascular Interventions*.

[4] Katritsis D. G., Siontis G. C. M., Ioannidis J. P. A. (2009). Double versus single stenting for coronary bifurcation lesions. *Circulation: Cardiovascular Interventions*.

[5] Hildick-Smith D., de Belder A. J., Cooter N. (2010). Randomized trial of simple versus complex drug-eluting stenting for bifurcation lesions. *Circulation*.

[6] Hahn J.-Y., Chun W. J., Kim J.-H. (2013). Predictors and outcomes of side branch occlusion after main vessel stenting in coronary bifurcation lesions: results from the COBIS II registry (COronary BIfurcation Stenting). *Journal of the American College of Cardiology*.

[7] Burzotta F., Trani C. (2015). Jailed balloon protection and rescue balloon jailing techniques set the field for safer bifurcation provisional stenting. *International Journal of Cardiology*.

[8] Burzotta F., Trani C., Sianos G. (2010). Jailed balloon protection: a new technique to avoid acute side-branch occlusion during provisional stenting of bifurcated lesions. Bench test report and first clinical experience. *EuroIntervention*.

[9] Singh J., Patel Y., Depta J. P. (2012). A modified provisional stenting approach to coronary bifurcation lesions: clinical application of the “Jailed-Balloon technique”. *Journal of Interventional Cardiology*.

[10] Depta J. P., Patel Y., Patel J. S. (2013). Long-term clinical outcomes with the use of a modified provisional jailed-balloon stenting technique for the treatment of nonleft main coronary bifurcation lesions. *Catheterization and Cardiovascular Interventions*.

[11] Çaylı M., Şeker T., Gür M. (2015). A novel-modified provisional bifurcation stenting technique: jailed semi-inflated balloon technique. *Journal of Interventional Cardiology*.

[12] Numasawa Y., Hase H., Yamazaki H. (2017). Three-dimensional optical frequency domain imaging of a true bifurcation lesion after stent implantation using the jailed semi-inflated balloon technique. *SAGE Open Medical Case Reports*.

[13] Thygesen K., Alpert J. S., Jaffe A. S. (2012). Third universal definition of myocardial infarction. *Journal of the American College of Cardiology*.

[14] Iakovou I., Schmidt T., Bonizzoni E. (2005). Incidence, predictors, and outcome of thrombosis after successful implantation of drug-eluting stents. *JAMA*.

[15] Colombo F., Biondi-Zoccai G., Infantino V. (2009). A long-term comparison of drug-eluting versus bare metal stents for the percutaneous treatment of coronary bifurcation lesions. *Acta Cardiologica*.

[16] Thuesen L., Kelbæk H., Kløvgaard L. (2006). Comparison of sirolimus-eluting and bare metal stents in coronary bifurcation lesions: subgroup analysis of the stenting coronary arteries in non-stress/benestent disease trial (SCANDSTENT). *American Heart Journal*.

[17] Zamani P., Kinlay S. (2011). Long-term risk of clinical events from stenting side branches of coronary bifurcation lesions with drug-eluting and bare-metal stents: an observational meta-analysis. *Catheterization and Cardiovascular Interventions*.

[18] Stinis C. T., Hu S. P. C., Price M. J., Teirstein P. S. (2010). Three-year outcome of drug-eluting stent implantation for coronary artery bifurcation lesions. *Catheterization and Cardiovascular Interventions*.

[19] Chaudhry E. C., Dauerman K. P., Sarnoski C. L., Thomas C. S., Dauerman H. L. (2007). Percutaneous coronary intervention for major bifurcation lesions using the simple approach: risk of myocardial infarction. *Journal of Thrombosis and Thrombolysis*.

[20] Kralev S., Poerner T. C., Basorth D. (2006). Side branch occlusion after coronary stent implantation in patients presenting with ST-elevation myocardial infarction. *American Heart Journal*.

[21] Saito S., Shishido K., Moriyama N. (2018). Modified jailed balloon technique for bifurcation lesions. *Catheterization and Cardiovascular Interventions*.

[22] Serruys P. W., Garcia-Garcia H. M., Onuma Y. (2012). From metallic cages to transient bioresorbable scaffolds: change in paradigm of coronary revascularization in the upcoming decade?. *European Heart Journal*.

[23] Diletti R., Tchetche D., Barbato E. (2016). Bioresorbable scaffolds for treatment of coronary bifurcation lesions: critical appraisal and future perspectives. *Catheterization and Cardiovascular Interventions*.

[24] van Geuns R. J., Gogas B. D., Farooq V., Regar E., Serruys P. W. (2011). 3-Dimensional reconstruction of a bifurcation lesion with double wire after implantation of a second generation everolimus-eluting bioresorbable vascular scaffold. *International Journal of Cardiology*.

[25] Seth A., Sengottuvelu G., Ravisekar V. (2014). Salvage of side branch by provisional “TAP technique” using absorb™ bioresorbable vascular scaffolds for bifurcation lesions: first case reports with technical considerations. *Catheterization and Cardiovascular Interventions*.

[26] Ruzsa Z., van der Linden M., Van Mieghem N. M. (2013). Culotte stenting with bioabsorbable everolimus-eluting stents. *International Journal of Cardiology*.

[27] van Mieghem N., Wilschut J. J., Ligthart J., Witberg K., van Geuns R.-J. M., Regar E. (2014). Modified T-technique with bioresorbable scaffolds ensures complete carina coverage. *JACC: Cardiovascular Interventions*.

